# Sepsis among Patients Admitted to the Intensive Care Unit of a Tertiary Care Centre

**DOI:** 10.31729/jnma.8275

**Published:** 2023-09-30

**Authors:** Laxman Mandal, Garima Rijal, Raj Singh, Bhawana Tiwari, Farhat Jahan, Dipti Lama, Sagun Shrestha, Ram Narayan Kurmi, Uma Das

**Affiliations:** 1 Department of Internal Medicine, Chitwan Medical College and Teaching Hospital, Bharatpur, Chitwan, Nepal; 2 Chitwan Medical College and Teaching Hospital, Bharatpur, Chitwan, Nepal; 3 Buddha Minimal Access Intervention Centre, Teku, Kathmandu, Nepal; 4 Himal Hospital, Gyaneshwor, Kathmandu, Nepal; 5 Maharajgunj Primary Health Centre, Maharajgunj, Kapilvastu, Nepal

**Keywords:** *infection*, *intensive care unit*, *sepsis*, *tertiary care centre*

## Abstract

**Introduction:**

Sepsis is a life-threatening dysfunction and is one of the common causes of admission in intensive care units. Early diagnosis and management improves the outcome of patients. The aim of this study was to find out the prevalence of sepsis among patients admitted to the intensive care unit of a tertiary care centre.

**Methods:**

A descriptive cross-sectional study was conducted among patients admitted to the intensive care unit of a tertiary care centre after obtaining ethical approval from the Institutional Review Committee. Data of patients admitted from 1 February 2022 to 31 January 2023 was collected between 6 April 2023 to 27 April 2023. Convenience sampling method was used. The point estimate was calculated at a 95% Confidence Interval.

**Results:**

Among 1001 patients, the prevalence of sepsis was 278 (27.77%) (25-30.54, 95% Confidence Interval). Among them, 209 (75.17%) developed septic shock. The mean age was 56.6±19.34 years. Pneumonia 43 (15.46%) and genitourinary infection 43 (15.46%) were the most common sources of infection and the source was unknown in 124 (44.60%) of patients. Hypertension 75 (26.97%) was the most common comorbidity. Acute kidney injury 166 (59.71%) was the most common complication followed by thrombocytopenia 165 (59.35%) and transaminitis 79 (28.41%).

**Conclusions:**

The prevalence of sepsis among patients admitted to the intensive care unit was higher than other studies done in similar settings.

## INTRODUCTION

Sepsis is defined as life-threatening organ dysfunction caused by a dysregulated host response to infection.^1^ Sepsis is a major public health problem causing a huge economic burden.^2,3^ About 48.9 million incident cases and 11.0 million sepsis-related deaths were reported in 2017.^4^ It may cause multiple organ dysfunction.^5^

Early diagnosis and treatment of sepsis improve the outcomes in terms of morbidity and mortality.^3^ Comorbidities like hypertension, and diabetes mellitus may coexist.^2^ As in Nepal, there is a lack of adequate data on the burden of sepsis in the intensive care unit (ICU), which ultimately enhances healthcare delivery and saves lives.

The aim of this study was to find out the prevalence of sepsis among patients admitted to the ICU in a tertiary care centre.

## METHODS

This descriptive cross-sectional study was conducted at Chitwan Medical College and Teaching Hospital, Bharatpur, Chitwan, Nepal. All patients admitted to the ICU from 1 February 2022 to 31 January 2023 were included in our study and data was collected from hospital records between 6 April 2023 to 27 April 2023. Ethical approval was obtained from the Institutional Review Committee of the same institute (Reference number: CMC-IRC/079/080-104). Patients who were less than 18 years and readmitted patients with the same diagnosis were excluded. Convenience sampling method was used. The sample size was calculated by using the following formula:


$$\begin{array}{ll} \text{n} & ={\text{Z}}^{2}\times \frac{\text{p}\times \text{q}}{{\text{e}}^{\text{2}}}\\ & =1.96^{2}\times \frac{0.50\times 0.50}{0.04^{2}}\\ & =601 \end{array}$$

Where,

n = minimum required sample size
Z = 1.96 at 95% confidence interval (CI)
p = prevalence taken as 50% for maximum sample size calculation
q = 1-p
e = margin of error, 4%

The minimum required sample size was 601. However, the final sample size taken was 1001. Sepsis was defined as life-threatening organ dysfunction caused by a dysregulated host response to infection. Organ dysfunction was defined as an acute change in total Sequential Organ Failure Assessment (SOFA) score >2 points consequent to the infection. Septic shock was defined as, in the clinical construct of sepsis with persisting hypotension requiring vasopressors to maintain mean arterial pressure (MAP) >65 mm Hg and having a serum lactate level >2 mmol/L (18 mg/dL) despite adequate volume resuscitation.^1^

Data were entered in Microsoft Excel 2010 and analysed using IBM SPSS Statistics version 21.0. The point estimate was calculated at a 95% CI.

## RESULTS

**Figure 1:**
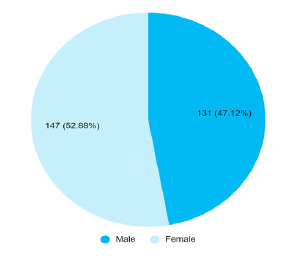
Gender-wise distribution of patients with sepsis (n= 278)

Among 1001 patients, 278 (27.77%) (25-30.54, 95% CI) developed sepsis. There were 147 (52.88%) females with a female to male ratio of 1.12:1 (Figure 1).

The patient's age ranges from 18-98 years with a mean age of 56.6±19.34 years. Most patients 64 (23.02%) lie within the range of 61-70 years (Table 1).

**Table 1:** Age-wise distribution of patients with sepsis (n= 278).

Age (years)	n (%)
<20	5 (1.79)
21-30	32 (11.51)
31-40	27 (9.71)
41-50	45 (16.18)
51-60	34 (12.23)
61-70	64 (23.02)
71-80	43 (15.46)
>80	28 (10.07)

In our study, the source of infection was unknown in 124 (44.60%) patients. Among the known sources of infection, pneumonia and genitourinary tract infection were present in 43 (15.46%) (Table 2).

**Table 2:** Sources of infection among patients with sepsis (n= 278).

Source of infection	n (%)
Unknown source	124 (44.60)
Pneumonia	43 (15.46)
Genitourinary infection	43 (15.46)
Gastrointestinal infection	27 (9.71)
Scrub typhus	26 (9.35)
Others	15 (5.39)

Among 278 patients, 167 (60.07%) had at least single underlying comorbidity whereas more than two comorbidities were present in 36 (12.94%) patients.

Hypertension 75 (26.97%) was the most common comorbidity followed by diabetes mellitus 54 (19.42%), chronic obstructive pulmonary disease (COPD) 46 (16.54%) and chronic liver disease (CLD) 33 (11.87%) (Table 3).

**Table 3:** Underlying comorbidity among patients with sepsis (n= 278).

Underlying condition	n (%)
Hypertension	75 (26.97)
Diabetes mellitus	54 (19.42)
COPD	46 (16.54)
CLD	33 (11.87)
Hypothyroidism	21 (7.55)
Coronary artery disease	10 (3.59)
Chronic kidney disease	7 (2.51)

Among 278 patients, 242 (87.05%) patients developed at least one complication. More than two complications were present in 110 (39.56%) patients. Besides shock, acute kidney injury (AKI) was the most common complication 166 (59.71%) followed by thrombocytopenia 165 (59.35%) and transaminitis 79 (28.41%). Electrolyte imbalance was present in 52 (21.48%) patients. Hypokalemia 25 (8.99%) was the most common electrolyte imbalance seen (Table 4).

**Table 4:** Complications among patients with sepsis (n= 278).

Complications	n (%)
AKI	166 (59.71)
Thrombocytopenia	165 (59.35)
Transaminitis	79 (28.41)
Multi-organ dysfunction syndrome	76 (27.33)
Hypokalemia	25 (8.99)
Acute respiratory distress syndrome	21 (7.55)
Hyponatremia	15 (5.39)
Respiratory failure	14 (5.03)
Metabolic acidosis	11 (3.95)
Hyperkalemia	8 (2.87)
Hypernatremia	4 (1.43)

Patients with sepsis and septic shock were managed with appropriate antibiotic and vasopressor. Vasopressor was used in 218 (78.41%) patients. Noradrenaline 216 (99.08%) was most commonly used but in some cases, dopamine 13 (5.96%) and vasopressin 12 (5.50%) were also needed. The maximum duration of ICU stay among patients with sepsis was 30 days with a mean duration of 4.22±3.66 days. Ventilator support was required in 92 (33.09%) patients. The mean duration of ventilation was 2.57±2.35 days. Out of 278 patients, 87 (31.29%) were discharged normally and 60 (21.58%) died during their hospital stay.

## DISCUSSION

In our study, the prevalence of sepsis was 278 (27.77%) which is higher as compared to other similar studies conducted in Singapor.^6^ About 52.88% of the study populations were female, having a female-to-male ratio of 1.12:1. A previous study conducted in Kathmandu, Nepal had a similar result.^7^ Patients' age ranges from 18 to 98 years with a mean age of 56.6±19.34 years, a previous study had a mean age of 51.56±19.48 years, which is quite similar to a previous study.^3^ Another study showed more than half of patients were above 60 years of age.^6^ Most patients 64 (23%) lie within the range of 61-70 years while a previous study showed 51-60 years of age.^3^

In our study, a total of 209 (75.17 %) patients developed septic shock which was corroborative with findings of a study conducted in Kathmandu.^7^ In our study, the source of infection was unknown in 124 (44.60%). Among the known sources of infection, pneumonia 43 (15.46%) and genitourinary infection 43 (15.46%) were the most common sources which was similar to other studies.^3,8^

Among 278 patients, 167 (60.07%) had at least a single underlying comorbidity whereas more than two comorbidities were present in 36 (12.94%) patients which is much lower than previous study conducted in Jordan, where about 62.2% of patients had at least 1-3 comorbidities.^9^ An observational previous study showed diabetes as the most prevalent comorbidities, which is 47.9%.^9^ In our study, hypertension was the most common comorbidity followed by diabetes mellitus and COPD. A similar finding was present in a study conducted in India.^5^

Out of 278 patients, 166 patients i.e. about 60% developed AKI followed by thrombocytopenia in 165 patients while multiple organ dysfunction was present in 76 patients (27.32%). A previous study also reported that 56.8% of patients developed AKI followed by cardiovascular system failure, and multiorgan failure was present in 21.9%.^10^

The mean duration of ICU stay among patients was 4.22±3.66 days which is similar to the finding of a previous study.^8^ In a previous study conducted in India, vasopressors were used in 25.50% of patients.^4^ Patients with sepsis and septic shock were managed with appropriate antibiotics and vasopressors. Vasopressor was used in 218 (78.41%) patients out of which noradrenaline 216 (99.08%) was most commonly used but in some cases, dopamine 13 (5.96%) and vasopressin 12 (5.50%) were also needed which is similar to the finding of a previous study.^11^

One study conducted in an ICU setting in India showed the need for ventilator support in 62.9% for a mean duration of 2.91±3.03 days.^5^ In our study, ventilator support was given to 92 (33.09%) patients. The mean duration of ventilation was 2.57±2.35 days.

A previous observational study showed that the mortality rate of patients with sepsis was 57.80% while another descriptive observational study in India showed a mortality rate of 42.2%.^9^ Out of 278 patients, 87 (31.29%) were discharged normally and 60 (21.58%) died during their hospital stay. In a previous study, the mortality rate was 36.5%, a similar study showed 35.2% and 39.3% mortality.^6,9,10^ The lower mortality rate in our study can be due to better medical service or many patients being taken home against medical advice.

There are also some limitations of the study as this is a single-centred study and might not be generalizable to a larger population.

## CONCLUSIONS

The prevalence of sepsis among patients admitted to the intensive care unit was higher than other studies done in similar settings. Recent evidence regarding risk stratification and prevention strategies should be dictated.
